# Quantifying thermoplastic mask quality for precision radiotherapy in head and neck cancer: A 3D stress test and 6D-axis error analysis

**DOI:** 10.1097/MD.0000000000040365

**Published:** 2024-11-01

**Authors:** Weirchiang You, Chienchih Chen, Hsiushan Chu, Yuchen Yau, Tingyang Liu, Peishuan Lai, Yungcheng Chen, Niwei Chen, Chefu Tsai, Heyuan Hsieh

**Affiliations:** a Department of Radiation Oncology, Taichung Veterans General Hospital, Taichung, Taiwan.

**Keywords:** head and neck cancers, radiotherapy, thermoplastic mask

## Abstract

Patients with head and neck cancers often require radiotherapy, where immobilization devices like thermoplastic masks ensure precise radiation delivery by minimizing movement. However, the quality of these masks lacks standard reference data. This study aimed to establish institutional acceptance criteria for thermoplastic mask quality and quantify their effectiveness using a 3 dimensional stress test and verified the setup errors using daily megavoltage computed tomography (MVCT). Between April and June 2022, 30 patients underwent radiotherapy with thermoplastic masks. Four key facial points (forehead, bilateral cheekbones, and chin) were tested for supporting force. Mean forces ranged from 3.97 N to 8.8 N. MVCT was used to assess 6 dimensional-axis errors, with mean translational errors (*x*, *y*, *z*) of 0.32 mm, −1.09 mm, and 2.24 mm, respectively, and rotational errors (yaw, pitch, roll) of −0.12°, 0.22°, and 0.35°, respectively. The results demonstrated that the thermoplastic masks provided precise immobilization, minimizing setup errors in 6 dimensions. Our findings offer a quantifiable method to ensure high-quality immobilization during radiotherapy for patients with head and neck cancers.

## 1. Introduction

There are more than 8000 newly diagnosed patients with head and neck cancers annually in Taiwan.^[1]^ The majority of patients with head and neck cancers had to receive the radiotherapy as part of the treatment modalities during their treatment course.^[2]^ There are several critical organs in the head and neck region, including cranial nerves, salivary glands, and internal carotid arteries, necessitating precise irradiation to effectively target tumors while sparing normal tissues and organs for patients with head and neck cancers. The accuracy of radiation delivery is closely associated with the patient’s daily treatment positioning and posture reproducibility.

The most commonly used immobilization device during radiotherapy sessions for patients with head and neck cancers is the thermoplastic mask, which ensures precise radiation delivery by minimizing patient’s movement. The pliable masks are made from thermoplastic sheets that conform to the contours of the patient’s anatomy from heated to cooled condition.^[3]^ There are several thermoplastic masks are developed as useful devices, such as the E-frame mask,^[4]^ 3 dimensional (3D)-printed mask,^[5,6]^ etc. In addition, several different positioning techniques have also been used to improve the precision and stability of the thermoplastic masks to fit different circumstances, such as head-tilted mask,^[7]^ minimal face and neck mask immobilization with optical surface guidance,^[8]^ Type-S (head only) mask (Civco) with head cushion/Uni-Frame mask (Civco) with head cushion, coupled with a BlueBag body immobilizer (Medical Intelligence)/Type-S head and shoulder mask with head and shoulder cushion (Civco)/same as previous, coupled with a mouthpiece,^[9]^ etc.

Despite these advances, the quality of the thermoplastic masks is difficult to quantify, and there are no established standard reference data. In current study, we provided our institutional acceptance criteria for thermoplastic mask quality, outline key steps in mask-making process, and perform a 3D stress test focusing on 4 specific facial points (forehead, bilateral cheekbones, and jaw). Additionally, we verify the precision of patient positioning errors using a 6 dimensional (6D)-axis evaluation with daily megavoltage computed tomography (MVCT) from Tomotherapy. Our goal is to standardize the supporting force of the thermoplastic mask at 4 key facial points and provide a quantifiable method to ensure a high-quality immobilization for patients undergoing head and neck cancer treatment.

## 2. Materials and methods

Between April and June 2022, 30 patients with head and neck cancers receiving curative radiotherapy (definite or postoperative adjuvant) were retrospectively enrolled. All patients underwent radiotherapy with a total dose of 60 to 70 Gy delivered over 30 to 35 fractions (once daily, 5 fractions per week) using Tomotherapy.

The thermoplastic masks used (BEACHCAST, T-TAPE COMPANY B.V. and Rolyan low temperature thernoplastics, OPC HEALTH) were subjected to the following acceptance criteria:

1. Verification of mold materials
Item names: ensure the mold names match the order.
Quantity: verify the number of molds corresponds to the order.
Warranty period: ensure the molds are within their warranty period.
2. Inspection of mold specifications
Outer packaging integrity: check for undamaged packaging.
Size specifications: confirm that the mold dimensions meet the procurement specifications.
Sharp edges or burrs: inspect the mold’s contact surfaces for any imperfections (sharp edges or burrs).
Misaligned or offset holes: ensure proper hole alignment.
Hole diameter: verify the holes are exantly 2 mm in diameter.
Thickness: confirm the mold thickness is 2.4 mm.
3. Sample testing of mold specifications
Testing and observation: test some molds for functionality (softening and forming). The standard settings of the softening are temperature 70 ± 2 °C in about 8 minutes. The residence time from softening to cooling of the mask on patient’s face is about 10 minutes.

**Figure 1.**
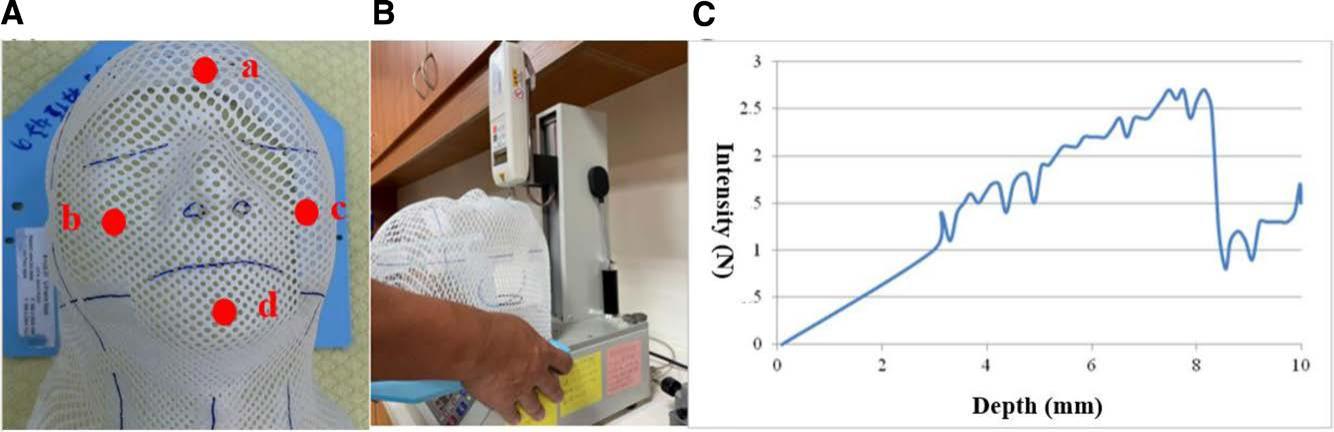
The facial points of the mask and 3D stress test. (A) Four facial points of the mask as (a) forehead, (b) right cheekbone, (c) left cheekbone, and (d) chin. (B) The 3D stress test. (C) The depth-intensity relationship diagram using 3D stress test.

Each patient’s thermoplastic masks were examined by 3D stress test (automatic servo stand handy force gauge, JSV-H1000 and digital & analog force gauge, ALGOL HF-100, Japan Instrumentation System, Nara, Japan). Four specific facial points were tested including forehead, bilateral cheekbones, and chin (Fig. 1A). To ensure accurate alignment throughout treatment and adequate coverage of primary cancer sites, such as carcinomas of the nasopharynx, oral cavity, oropharynx, and hypopharynx, 4 specific bony landmarks are selected. These landmarks are chosen for their stable positions, which remain consistent despite changes in soft tissue over time. These points were pressed to a depth of 1 cm and the intensities (N, newton) were recorded, respectively (Fig. 1B, C).

Patients received daily image-guided radiotherapy (IGRT) using MVCT imaging, and 6D-axis errors (*x*, *y*, *z*, yaw, pitch and roll) before machine correction were documented throughout the treatment.

## 3. Results

Thirty patients were reviewed and the tumor locations were nasopharyngeal cancer in 19 patients, oral cavity cancer in 7 patients and oropharyngeal cancer in 4 patients, respectively. Most patients (50%) received definite concurrent chemoradiotherapy and only 4 patients received radiotherapy alone without any chemotherapy. Four patients suffered from grade 3 radiation mucositis and 4 patients experienced grade 3 radiation dermatitis, respectively. The patients’ characteristics were shown in Table 1.

**Table 1:** Patients’ characteristics.

Age (median [range]) (yr)	51.5 (36–73)
Gender (M/F)	23/7
Primary tumor location (%)	
Nasopharynx	19 (63.3)
Oral cavity	7 (23.3)
Oropharynx	4 (13.3)
Dose and fractionation (%)	
70 Gy in 35 fractions	21 (70.0)
66 Gy in 33 fractions	5 (16.7)
60 Gy in 30 fractions	4 (13.3)
Treatment (%)	
CCRT	15 (50.0)
RT alone	4 (13.3)
Induction chemotherapy + RT alone	8 (26.7)
Induction chemotherapy + CCRT	3 (10.0)

CCRT = concurrent chemoradiotherapy, RT = radiotherapy.

Forty thermoplastic masks were tested (including pretreatment initial thermoplastic masks with or without 2nd masks according to changes in patients’ facial shape). The mean forces and the standard deviation (SD) recorded at the 4 points using 3D stress test were:

Forehead: 3.97 N (SD 3.10)
Right cheekbone: 4.32 N (SD 2.97)
Left cheekbone: 4.62 N (SD 3.29)
Chin: 8.8 N (SD 5.9)

A total of 920 MVCT images were analyzed and the 6D-axis errors before machine correction were:

*x*: 0.32 mm (SD 1.22)
*y*: −1.09 mm (SD 1.63)
*z*: 2.24 mm (SD 1.72)
yaw: −0.12° (SD 0.34)
pitch: 0.22° (SD 0.29)
Roll: 0.35° (SD 0.38)

## 4. Discussion and conclusion

Radiation therapy plays a critical role in the treatment of head and neck cancers. However, the complexity of this anatomical region, which houses several vital organs and tissues, poses significant challenges. The proximity of critical structures, such as the brainstem, spinal cord, optic nerves, carotid arteries, and salivary glands, makes it difficult to deliver adequate radiation to the tumor without risking damage to these sensitive areas. Therefore, precise delivery of radiation is essential to ensure that therapeutic doses effectively target the tumor while minimizing exposure to normal tissues and critical organs. This precision not only maximizes treatment efficacy but also reduces the risk of side effects and long-term complications for patients.

One of the key factors in achieving precise radiation delivery is ensuring reproducibility during each treatment session. Patients with head and neck cancers typically undergo radiotherapy in multiple sessions over several weeks, and any variation in positioning from 1 session to another can lead to suboptimal targeting of the tumor and potential harm to surrounding tissues. Reproducibility can be affected by several factors, including patient movement, the accuracy of immobilization devices, and the quality of the positioning setup. Thus, minimizing setup errors, particularly in 6 dimensions (the 3 translational axes [*x*, *y*, *z*] and 3 rotational axes [pitch, yaw, and roll]) is paramount for successful radiotherapy outcomes.

Most studies aimed at improving precision in radiotherapy for patients with head and neck cancers have concentrated on optimizing immobilization devices and patient treatment positioning.^[4–9]^ Immobilization devices, such as thermoplastic masks, are widely used to help patients remain still during radiation therapy. These masks are custom-fitted to each patient’s facial contours and securely attach to the treatment table to reduce movement. The goal of these devices is to achieve the most consistent and stable patient position possible, session after session, thereby minimizing deviations in the delivery of the radiation dose. Various immobilization devices have been evaluated for their effectiveness in reducing setup errors. For example, studies have compared different types of thermoplastic masks, such as Type-S masks and E-Frame masks,^[4]^ and explored different patient positioning techniques. These studies have yielded promising improvements in reducing errors in the translational (*x*, *y*, *z*) and rotational (pitch, yaw, roll) axes. However, while significant progress has been made in these areas, little attention has been given to the quality and production process of the thermoplastic masks themselves, which could further enhance treatment accuracy.

Our study is the first study to investigate and quantify the quality of the thermoplastic masks using a 3D stress test on 4 facial points of the mask to evaluate their effectiveness in minimizing 6D-axis errors during radiotherapy for patients with head and neck cancers. In our analysis, we focused on 4 key points of the mask: the forehead, bilateral cheekbones, and chin, which are critical in securing the mask to the patient’s face and minimizing movement during treatment. By using daily MVCT scans, we were able to assess the corresponding setup errors in all 6 dimensions. Our findings revealed that the pretreatment thermoplastic masks performed well in terms of reducing setup errors. The mean 6D-axis errors in our study were satisfactory and comparable to those reported in previous research. For example, the translational errors in the *x*, *y*, and *z* axes for the Type-S mask were 1.5, 3.1, and 2.7 mm, respectively, while for the E-Frame mask, they were 1.4, 2.5, and 2.8 mm, respectively.^[4]^ Our study produced similar results for these axes, indicating that the quantified thermoplastic masks provide comparable levels of precision in translational positioning. Rotational errors (pitch, yaw, and roll) can be particularly challenging to control, as even slight variations in rotation can result in significant misalignment of the radiation beam. A study by Velec et al^[10]^ demonstrated that the interfraction rotational errors for standard thermoplastic masks were 0.8° in each of the 3 axes using cone beam computed tomography (CBCT). In our current study, we observed mean errors of 0.22° for pitch, −0.12° for yaw, and 0.35° for roll, which represent an improvement and non-inferior outcomes compared to previous findings. These small rotational errors suggest that our quantified thermoplastic masks are effective in reducing setup variability and ensuring consistent patient positioning across multiple treatment sessions.

IGRT has emerged as a powerful tool for improving the precision of daily radiation delivery. IGRT involves using imaging techniques, such as CBCT, to verify patient positioning before each radiation session and make adjustments if necessary. This ensures that the radiation is delivered to the exact location of the tumor, even if the patient’s position has slightly shifted from the previous session. While IGRT offers significant benefits in terms of treatment accuracy, it also comes with additional costs. Patients must undergo daily imaging, which can increase the overall cost of treatment. Unfortunately, many patients with head and neck cancers face financial difficulties and may be unable to afford the cost of daily IGRT. Socioeconomic factors play a significant role in determining access to advanced cancer treatments, and patients from lower socioeconomic backgrounds are often unable to take advantage of the latest technologies. This financial barrier can limit their ability to receive the most precise and effective radiation therapy available.

Despite the challenges associated with limited access to IGRT, our study demonstrates that patients can still receive high-quality and precise radiotherapy without the need for daily image correction. By quantifying and optimizing the quality of the thermoplastic masks, we have shown that it is possible to minimize setup errors and maintain accurate patient positioning throughout the course of treatment. This approach allows patients to benefit from precise radiation delivery even if they are unable to afford daily imaging. Our study provides an important step forward in ensuring equitable access to high-quality cancer treatment. By focusing on the production and quality of thermoplastic masks, we offer a cost-effective solution for reducing setup errors and improving treatment accuracy. This is particularly important for patients with head and neck cancers, where precision is crucial to avoid damage to critical structures and ensure the best possible outcomes.

In conclusion, this study established quantifiable methods to assess the quality of thermoplastic masks used in radiotherapy for patients with head and neck cancers. By applying a 3D stress test to key facial points and analyzing daily 6D-axis positioning errors using MVCT, we demonstrated the measured forces at critical points on the mask can provide effective immobilization with minimized setup errors in 6 dimensions. These findings offer a practical and cost-effective approach to ensuring consistent, high-quality radiotherapy without the need for daily image correction, making it more accessible for patients facing financial constraints. Overall, standardizing the supporting force of thermoplastic masks can significantly enhance treatment accuracy and improve patient outcomes.

## Author contributions

**Conceptualization:** Weirchiang You, Chienchih Chen, He-Yuan Hsieh.

**Data curation:** Hsiushan Chu, Yuchen Yau, Tingyang Liu, Peishuan Lai, Yungcheng Chen, Niwei Chen, Chefu Tsai, He-Yuan Hsieh.

**Formal analysis:** Niwei Chen, He-Yuan Hsieh.

**Investigation:** He-Yuan Hsieh.

**Methodology:** Hsiushan Chu, Peishuan Lai, Yungcheng Chen, He-Yuan Hsieh.

**Software:** Niwei Chen, He-Yuan Hsieh.

**Supervision:** He-Yuan Hsieh.

**Validation:** Weirchiang You, Chienchih Chen.

**Visualization:** Weirchiang You, Chienchih Chen, He-Yuan Hsieh.

**Writing – original draft:** He-Yuan Hsieh.

**Writing – review & editing:** Weirchiang You, Chienchih Chen.
